# Affinity microfluidics enables high-throughput protein degradation analysis in cell-free extracts

**DOI:** 10.1038/s42003-022-04103-3

**Published:** 2022-10-28

**Authors:** Lev Brio, Danit Wasserman, Efrat Michaely-Barbiro, Gal Barazany-Gal, Doron Gerber, Amit Tzur

**Affiliations:** The Mina & Everard Goodman Faculty of Life Sciences and the Institute for Nanotechnology and Advanced Materials, Bar Ilan University, Ramat Gan, Israel

**Keywords:** Lab-on-a-chip, Proteomic analysis

## Abstract

Protein degradation mediated by the ubiquitin-proteasome pathway regulates signaling events in many physiological and pathological conditions. In vitro degradation assays have been instrumental in the understanding of how cell proliferation and other fundamental cellular processes are regulated. These assays are direct, time-specific and highly informative but also laborious, typically relying on low-throughput polyacrylamide gel-electrophoresis followed by autoradiography or immunoblotting. We present protein degradation on chip (pDOC), a MITOMI-based integrated microfluidic technology for discovery and analysis of proteins degradation in cell-free extracts. The platform accommodates hundreds of microchambers on which protein degradation is assayed quickly, simultaneously and using minute amounts of reagents in one or many physiochemical environments. Essentially, pDOC provides a sensitive multiplex alternative to the conventional degradation assay, with relevance to biomedical and translational research associated with regulated proteolysis.

## Abstract (web summary)

A protein degradation on chip technology enables high-throughput discovery and analysis of ubiquitin proteasome pathway-regulated protein degradation in cell-free extracts.

## Introduction

Protein degradation by the ubiquitin-proteasome system is a central regulatory module through which the level of proteins in all eukaryotic cells remains balanced. Deviation from the desired amount of each protein at any given moment can be detrimental to the cell, leading to dysfunctional tissues and a wide range of diseases in human, including cancer, cystic fibrosis, and neurodegenerative disorders^1,2^.

The core cascade underlying ubiquitination involves three enzymes: The E1 enzyme covalently binds and activates the ubiquitin molecule for transfer to an E2 conjugating enzyme. Then, the ubiquitin-conjugated E2 interacts with an E3 ubiquitin-ligase enzyme, which catalyzes the transfer of ubiquitin molecules from the E2 to the target protein, typically via an isopeptide bond to a lysine residue. Repetitive ubiquitination events can form one or more chains of ubiquitin moieties on the target protein (i.e., Polyubiquitination). A polyubiquitin chain that is recognized by the proteasome triggers protein degradation^1,2^. Hundreds of different E3 enzymes govern the enormous functional reach and specificity of the entire ubiquitination process. With respect to cell proliferation and cell cycle regulation, the ubiquitin ligases anaphase-promoting complex/cyclosome (APC/C) and Skp1-Cullin-F-box protein complex (SCF) are particularly important^3–5^. The substrate specificity of both complexes is dependent on co-activators: Cdc20 and Cdh1 for the APC/C and one of several F-box proteins for the SCF, e.g., Skp2 and β-TrCP^6–8^. Overall, orderly proteolysis mediated by cell cycle-regulated E3 enzymes ensures unidirectional cell cycle in all eukaryotes^6–10^.

Whereas some forms of ubiquitin chains marks proteins for degradation by the proteasome, monoubiquitination and other forms of polyubiquitination regulate signaling cascades via proteasome-independent pathways^11^. Furthermore, ubiquitination can be reversed by enzymes called deubiquitinases in a manner that can prevent proteolysis^12^. On the flip side, proteasomal degradation may not always be coupled to ubiquitination^13^. Thus, protein degradation cannot be inferred from ubiquitination and must be determined directly.

Protein degradation assays in cell-free extracts, also known as ‘cell-free systems’, have been instrumental in cell biology research, enabling direct and quantitative analyses of ubiquitin-mediated proteolysis in physiologically relevant environments. In fact, much of the cell cycle principles were discovered by monitoring the degradation of cell cycle proteins in extracts from frog eggs or cycling human cells (see for example^14–20^). The extensive use of these ‘degradation assays’ in today’s modern era, is a testament to their efficacy (see for example^21–23^). Interestingly, conventional degradation assays have never truly benefited from modern technologies, and still rely on gel-electrophoresis, autoradiography or immunoblotting, and large amount of biological material.

Integrated microfluidics and pneumatic microvalves paved the way to protein chips in which arrayed proteins are freshly expressed in reticulocyte lysates that maintain proper protein folding and activity^24^. The target proteins are expressed either on-chip or externally, and subsequently immobilized to microchambers via a designated surface chemistry. Then, a large panel of fluorometric assays can be performed over thousands of microchambers, using minute amounts of reagents. In recent years, we developed several applications based on mechanically induced trapping of molecular interactions (MITOMI), for multiplexed in situ analysis of protein post-translational modifications (PTMs) and noncovalent interactions^24–30^.

The combination of integrated microfluidics, protein arrays and cell-free systems holds great potential in biomedical research. In this study, we demonstrate a MITOMI-based platform for the detection and quantification of protein degradation in cell-free extracts. The method, named ‘pDOC’ (protein degradation on chip), provides a fast, multiplex alternative to the classic method by which proteasome-mediated proteolysis has been assayed in vitro almost unvaryingly for nearly half a century.

## Materials and method

### Plasmids

pCS2-Flag-FA vector was generated by annealing Flag tag oligos and ligating final fragment into pCS2-FA vector using BamHI and FseI restriction enzyme (RE) sites. pCS2-Flag-FA-Securin-GFP w.t and ∆64 variant plasmids were generated by cloning w.t or ∆64 Securin-GFP^27^ into pCS2-Flag-FA vector, using FseI (5’) and AscI (3’) flanked primers. The plasmid pCS2-FA-Geminin-GFP has been described^27^. pCS2-FA-Geminin∆27-GFP was generated by amplifying the open reading frame (ORF) of Geminin-GFP starting at the codon for Methionine 28 using FseI (5’) and AscI (3’) flanked primers. The PCR product was recloned into the pCS2-FA vector. The plasmids pCS2-Flag-FA-Geminin-GFP w.t and ∆27 variant were generated by cloning each of the two Geminin-GFP variants into pCS2-Flag-FA vector using FseI and AscI RE sites. Flag-p27-myc fragment was generated by a two-step assembly PCR using pCS2-Flag-FA-p27-GFP template, a first primer set containing a Flag tag (5’) and a Myc tag (3’), and a second primer set containing a T7 promoter (5’) and a T7 terminator sequence (3’). Geminin N-terminus (110 amino acids) attached to monomeric Azami-Green (mAG) was amplified using a previously described template^31^ and a primer set flanked with FseI (5’) and AscI (3’) RE sites. The PCR product was cloned into pcS2FA-FLAG plasmid. The latter was used as a template to generate a ∆27 variant by mutagenesis (Agilent’s QuikChange® Lightning kits; 210513). K-to-A and RxxL to GxxV substitutions (single amino acid code) were generated by mutagenesis. See supplementary Table 1 for more details. Aside from mAG-Geminin, all GFP-tagged proteins caried enhanced variant of green fluorescent protein (eGFP). See Supplementary Table 1 for oligo sequences.

### Cell culture maintenance

NDB cells are based on the HEK293 cell line. A detailed description of this cell system can be found in ref. ^17^. NDB and HeLa S3 (ATCC; #CCL-2.2) cells were maintained in tissue culture dishes containing Dulbecco’s Modified Eagles Medium (DMEM) supplemented with 10% fetal bovine serum, 2 mM L-glutamine, and 1% Penicillin–Streptomycin solution (Biological Industries; #01-055-1A, #04-001-1A, #03-020-1B, #03-031-1B). Cells were maintained at 37 °C in a humidified 5% CO_2_-containing atmosphere. HeLa S3 cells were either cultured on dishes or in 1-l glass spinner flasks in suspension (80 rpm). NDB cells were cultured in the presence of 5 μg/ml Blasticidin (Life Technologies; #A11139-03) to maintain the pcDNA6/TR plasmid carrying the ORF for Tet repressor.

### Cell synchronization

For late-mitosis synchronization, NDB cells were cultured in 150 mm/diameter dishes. After reaching a confluency of about 75%, the cells were treated with 1 μg/ml Tetracycline (Sigma-Aldrich; #87128) for 22 h and harvest for extract preparation. For S-phase synchronization, HeLa S3 cells were cultured in suspension for 72 h up to a concentration of approximately 5 × 105 cells/ml. Cells were then supplemented with 2 mM Thymidine for 22 h, washed with DMEM (twice, 5 min, 250 × *g*) and released into pre-warmed fresh media (37 °C) for additional 9 h. Cell culture was then supplemented again with 2 mM Thymidine for 19 h before harvest for extract preparation.

### Preparation of cell extracts

HeLa S3 extracts: S-phase Synchronous HeLa S3 cells were washed with ice-cold 1× PBS and lysed in a swelling buffer (20 mM HEPES, pH 7.5, 2 mM MgCl_2_, 5 mM KCl, 1 mM Dithiothreitol [DTT], and protease inhibitor cocktail [Roche; #11836170001]) supplemented with energy-regenerating mixture, E-mix (1 mM ATP, 0.1 mM ethylene glycol-bis [β-aminoethyl ether]-N,N,N′,N′-tetra acetic acid [EGTA], 1 mM MgCl_2_, 7.5 mM creatine phosphate, 50 μg/ml creatine phosphokinase). Cells were incubated on ice for 30 min and homogenized by freeze-thawing cycles in liquid nitrogen and passed through a 21-G needle for 10 times. Extracts were cleared by subsequent centrifugation (17,000 × g; 10 and 40 min), and stored at –80 °C. NDB mitotic extracts: Tet-induced NDB cells were collected from 20–24 150 mm dishes by gentle wash with ice-cold PBS. Extracts were prepared as described above for HeLa S3 cells. For more details see^17,32^.

### In vitro expression of target proteins

Target proteins were in-vitro expressed using rabbit reticulocyte lysate (TNT-coupled reticulocyte system; Promega; #L4600, #L4610) supplemented with either ^35^S-methionine/S-L-cysteine mix (PerkinElmer; #NEG772002MC) for radiography detection or with untagged Methionine (Promega #L118A) and FluoroTect™ GreenLys (Promega #L5001).

### Off-chip degradation assay

Degradation assays were performed in 20 μl cell extract supplemented with 1 μl of 20× energy regenerating mixture (see above), 1 μl of 10 mg/ml Ub solution (Boston Biochem; #U-100H), and 1 μl radiolabeled in vitro translated protein of interest. For a negative control, reaction mixture was supplemented with proteasome inhibitor MG132 (20 μM; Boston Biochem; #I-130). Reaction mixtures were incubated at 28 °C, and samples of 4–5 µl were collected in 15–20 min intervals. Off-chip detection: Time-point samples were mixed with 4× Laemmli Sample Buffer (BIO-RAD #1610747), denaturized (10 min, 95 °C), and resolved by SDS-PAGE. Gels were soaked in a Methanol/Acetic acid (10/7.5%) fixative solution for 20 min, dried in vacuum and heat, and exposed to phosphor screen (Fuji) for 24–72 h. In vitro translated proteins were visualized by autoradiography using Typhoon FLA 9500 Phosphorimager (GE Healthcare Life Sciences). Signal intensity (corrected for background signal) was measured by ImageJ software and was normalized to the signal at *t*_0_. All plots were created using Microsoft Excel software, version 16.20. Mean and SE values were calculated from three or four independent degradation assays. On-chip detection: Time-point samples were immediately frozen in liquid nitrogen. Before detection, samples were thawed on ice, flowed through the chip 5 min, and immobilized to protein chambers under the ‘button’ valve (see ‘Surface chemistry’ below). Next, the ‘button’ valves were closed, allowing unbound material to be washed by PBS. The level of target proteins (before and after degradation reactions) were determined by 488 nm-excitation and an 535/25 nm emission filter. Protein level could also be measured by immunofluorescence using fluorescently labeled antibodies (anti-Flag-Alexa 647, μg/ml, #15009; Cell Signaling, Danvers, MA, USA). These antibodies were flowed into the device and incubated with the immobilized proteins under the ‘button’ for 20 min at RT. Unbound antibodies were mechanically washed by PBS following the closing of the ‘button’ valve. Here, target protein levels were determined by 633 nm-excitation and an 692/40 nm emission filter.

### Device fabrication and activation

The microfluidic device is made of two layers of PDMS. The silicon wafers are written by photolithography (Heidelberg MLA 150). Then after, the soft lithography phase is performed using silicon elastomer polydimethylsiloxane (PDMS, SYLGARD 184, Dow Corning, USA) and its curing agent to fabricate the microfluidic devices. The microfluidic devices are consisting of two aligned PDMS layers, the flow and the control layers which are prepared using different ratios of PDMS and its curing agent; 5:1 and 20:1 for the control and flow layers, respectively. The control layer is degassed and baked for 30 min at 80 °C. The flow layer is initially spin-coated (Laurell, USA) at 2000 rpm for 60 s and baked at 80 °C for 30 min. Next, the flow and control layers are aligned using an automatic aligner machine (custom made) under a stereoscope and baked for 1.5 h at 80 °C for final bonding. The two-layer device is then peeled off from the wafer and bound to a cover slip glass via plasma treatment (air, 30%, 30 s). While operating, the device is connected to a pneumatic manifold, using Tygon® tubing and is automatically operated based on programmed set of commands.

### Surface chemistry

Biotinylated-BSA (1 μg/μl, Thermo) is flowed for 25 min through the device, allowing its binding to the epoxy surface. On top of the biotinylated-BSA, 0.5 μg/μl of NutraAvidin (Pierce, Rockford, IL) is added (flow for 20 min). The ‘button’ valve is then closed, and biotinylated-PEG (1 μg/μl, (PG2-AMBN-5k, Nanocs Inc.) is flowed over for 20 min, passivating the flow layer, except for the buttons area. Following passivation, the ‘button’ valve is released and a flow of 0.2 μg/μl biotinylated anti-GFP antibodies (Abcam; #ab6658, Cambridge, United Kingdom) or 0.01 µg/µl biotinylated anti-Flag antibodies (Cell Signaling; #2908 S Danvers, MA, USA) were applied. The antibodies bound to the exposed NutraAvidin, specifically to the area under the ‘button’, creating an array of anti-GFP - or anti-Flag tag. PBS buffer was used for washing in between steps. In the case of p27 immobilization, surface chemistry was performed with 0.2 μg/ml donkey anti-mouse whole biotinylated anti-IgG antibodies (#715-065-150, Jackson Immuno research laboratories, Maryland, USA) followed by 20 min flow of 6.5 μg/ml anti p27 antibodies (Santa Cruz biotechnology, Heidelberg Germany; #1641 mouse).

### On-chip degradation assay

Flag-Securin-GFP (w.t and ∆64 mut) and p27-GFP IVT products were flowed into the chip and immobilized on the surface under the ‘button’ at the protein chambers via its GFP tag, following by PBS buffer wash and scanned. Next, the ‘button’ valves were opened and the extract reaction mixtures were incubated with the protein chambers for 60 min (30 °C). During the reaction, the level of the remining target protein was determined by GFP signal every 15 min. The decline in GFP signal correlated with degradation. After background signal subtraction, GFP signals were normalized to the signal at *t*_0_ (value of 1) or between 1 (max signal) and 0 (min signal).

### Image and data analysis

LS reloaded microarray scanner, GenePix7.0 (Molecular Devices) and ImageJ image analysis software were used for analysis and presentation of the images. The signal measured around the button valve was considered as the background, since no immobilization of proteins was expected there. Yet, some background signal is always detected, which results from nonspecific attachment of antibodies to the device surface. We subtracted the background signal around the buttons in a ring the size of 2 R with 2-pixel spacing (see supplementary material in^27^).

### Immunoblotting

Protein samples were mixed with x4 Laemmli buffer, denatured (10 min, 96 °C), and resolved on freshly made 10% acrylamide gel using a Tris-glycine running buffer. Proteins were then electro-transferred onto a nitrocellulose membrane (Bio-Rad; #162-0115) using Trans-Blot Turbo transfer system (Bio-Rad). Ponceau S Solution (Sigma-Aldrich; #81462) was used to verify transfer quality. Membrane was washed (TBS), blocked (5% skimmed milk in TBST), and incubated (RT, 1 h) with antibody solution (2.5% BSA and 0.05% sodium azide in PBS) before blotted with anti-Securin (Abcam; #AB3305) primary antibody (RT, 2 h). Anti-mouse Horseradish peroxidase-conjugated secondary antibody was purchased from Jackson ImmunoResearch (#115-035-003). ECL Signal was detected using EZ-ECL (Biological Industries; #20-500-171).

### Statistics and reproducibility

Overall, details about experimental design and statistics are given in the relevant sections. *P* values were calculated based on two-tailed unpaired *t*-test. In gel analyses performed in triplicates; plots depict mean and standard error (SE) values. Statistics for On-chip analysis were derived from *n* = 10 to 57 cell unites. Mean and SE values are shown. Statistical analysis and graphs were generated using Microsoft Excel v16.65 software.

## Results

**Fig. 1:**
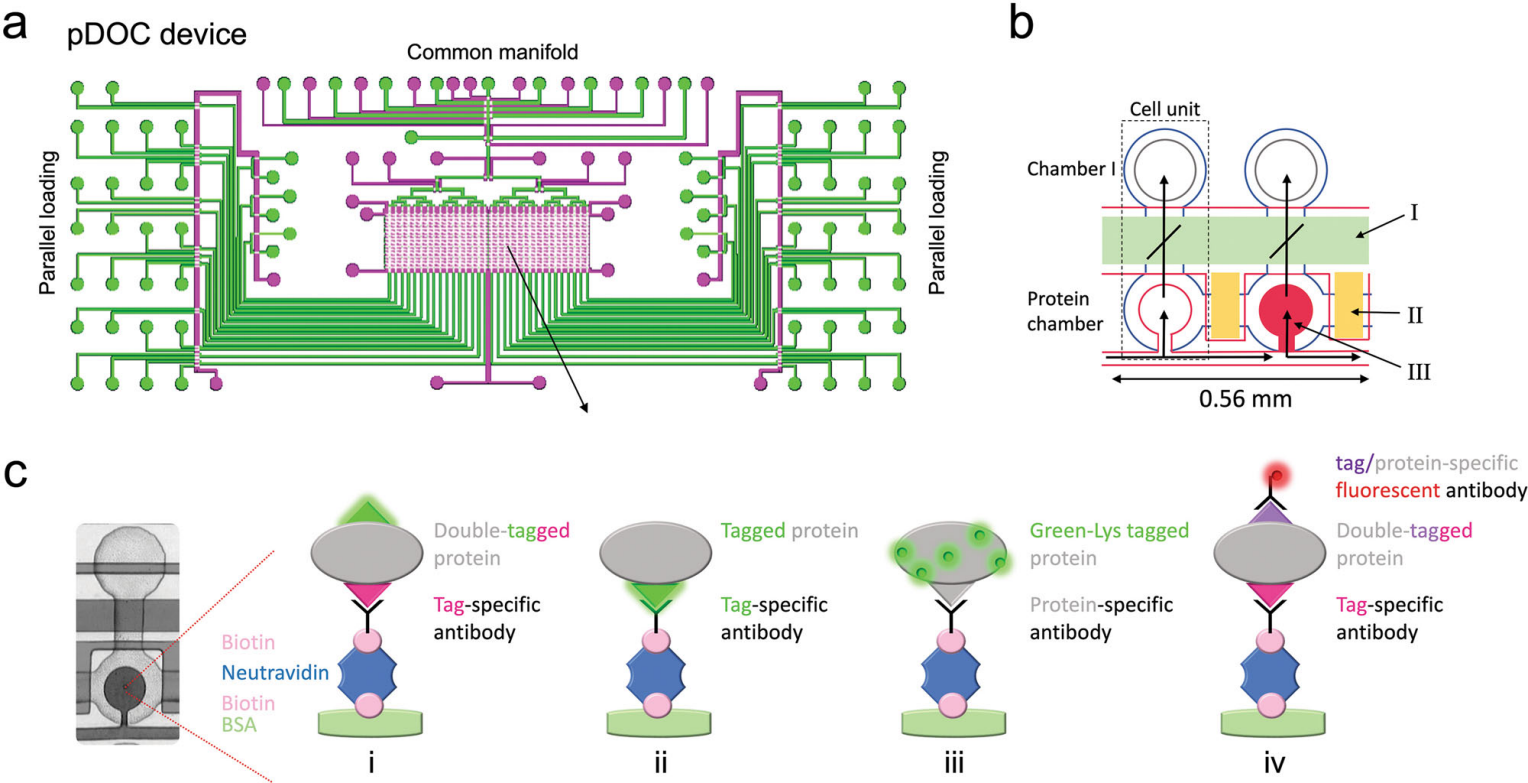
Design and strategy toward protein degradation assay on-chip. **a** pDOC is a MITOMI-based microfluidic platform. The device includes flow (green) and control (Magenta) layers, and several modules: 1) common manifold enabling loading of materials, which in combination with microarray can enable up to 512 different experiments; 2) Parallel loading inputs enabling gel-like loading of up to 32 different experimental conditions; and 3) MITOMI module of 512 cell units, which controls the degradation assay process. **b** An illustration of two cell units. Each cell unit (marked by black dotted frame) comprises two chambers controlled by three pneumatic integrated valves. The total volume of each unit is about 1 nanoliter. The two chambers are separated by the ‘neck valve’ (I). Different cell units are separated via sandwich valves (II). Samples containing target proteins (IVT products) are loaded into the protein chamber and immobilized via biotinylated antibodies that can be either protein- or tag specific. The target proteins are trapped via MITOMI button valve (III) for quantification while the remaining unbound biomaterials are washed away. Flow direction within the device is indicated by green arrows. **c** Left, an image of a cell units within the MITOMI array. Target proteins can be immobilized to the protein chamber and quantified in several ways (illustrated on the right): i) An example of a target protein carrying a fluorescent tag (e.g., GFP) for detection (see green glowing tag) and a non-fluorescent tag for immobilization; ii) A target protein tagged with GFP for both immobilization (by anti-GFP antibodies) and detection; iii) The target protein is untagged. Detection is based on fluorescent lysine (Lys) incorporated during in vitro translation. Immobilization is via protein-specific antibodies. iv) The target protein is immobilized via tag- or protein-specific antibodies and detected by immunofluorescence via tag-specific antibodies coupled to fluorophore. Overall, on-chip immobilization of target proteins relies on biotinylated antibodies. Immobilization via non-biotinylated antibodies is possible if surface chemistry includes biotinylated IgG.

We designed pDOC to support multiple strategies for analyzing protein degradation in vitro, specifically cell-free systems. The device is based on a MITOMI integrated microfluidic chip, originally developed to quantify protein-ligand interactions at equilibrium^24,33^. The MITOMI module was modified to contain an array of 16 × 64, 32 × 32 or 32 × 16 microcompartments. The latter chip was also devised to enable parallel loading (see schematics in Fig. 1a). Each compartment, i.e., cell unit (Fig. 1b), is separated into two chambers and controlled by three valves: a ‘neck’ valve that controls the diffusion (mixing) of material from chamber I into the ‘Protein chamber’, in which a specific target protein is trapped; a ‘sandwich’ valve that separates between cell units; and the MITOMI ‘button’ valve, which traps interacting molecules beneath it, thus taking a snapshot of the interaction at equilibrium (Fig. 1b). Chamber I can be loaded in one of two ways: 1) “Gel-like” loading, which enable sequential or parallel loading of 32 samples. This is performed by an array of individual input channels controlled by micro-valves. The advantage of this approach is the shorter time to results, its relative simplicity, and the conceptual similarity to conventional gel-based protocols. 2) Microarray loading, i.e., pre-spotted open reading farmes (ORFs) are transcribed on-chip to generate an array of freshly expressed proteins. This approach scales up throughput. No less important, it scales down reagent consumptions by at least 3 orders of magnitude compared to the conventional assay. Each reaction is less than a nanoliter in volume. The common manifold is then used to load common materials to the different sections of the device (e.g., for surface chemistry). Furthermore, pDOC chip designs include the separation of the master control of the three valves into sections of the chip that can be activated and controlled independently. Compared to the control of each valve type for the entire chip, this separation not only improves valve response, but more importantly, it permits time-response assays on chip. Within each section, experiments can be performed in all cell units in parallel, enabling multiplexed applications of the kind shown in our previous devices^24–26,34^.

Like the classic degradation assays, target proteins for pDOC analyses are in vitro translated (IVT) using rabbit reticulocyte lysate that allows correct folding and post-translational modifications (PTMs). The IVT products are immobilized on the glass surface of the chip via biotin-avidin binding. To this end, specific biotinylated antibodies are applied under the button valve. Following pull down of the target protein the unbound material, such as reticulocyte lysate and cell extract, is washed away. Then the entire chip is passivated by PEG-Biotin, except for the area beneath the button (Fig. 1b). The bound target proteins are quantified fluorometrically. For more details, see our previous publications^25,26,34^.

The device is compatible with multiple strategies of surface chemistry and possible experimental setups (illustrated in Fig. 1c): 1) The target protein is tagged on both N’ and C’ termini. One tag is used for immobilization via tag-specific biotinylated antibodies and the second tag is a fluorescent protein (e.g., GFP), which is used for detection. 2) A fluorescent protein is used for both immobilization and detection. 3) The target protein is translated in lysate containing fluorescent lysine (green-Lys) and immobilized by protein-specific antibodies. 4) The target protein is double tagged. Here, however, one tag is used for immobilization whilst the second tag is immunodetectable by the fluorescent antibody. Importantly, immobilization can be performed with non-biotinylated antibodies if the surface chemistry also includes biotinylated anti IgG. This flexibility of protein immobilization and quantification simplifies assay optimization according to specific needs and limitations, and the overall versatility of the platform.

### pDOC facilitates analysis of protein degradation in cell-free extracts

**Fig. 2:**
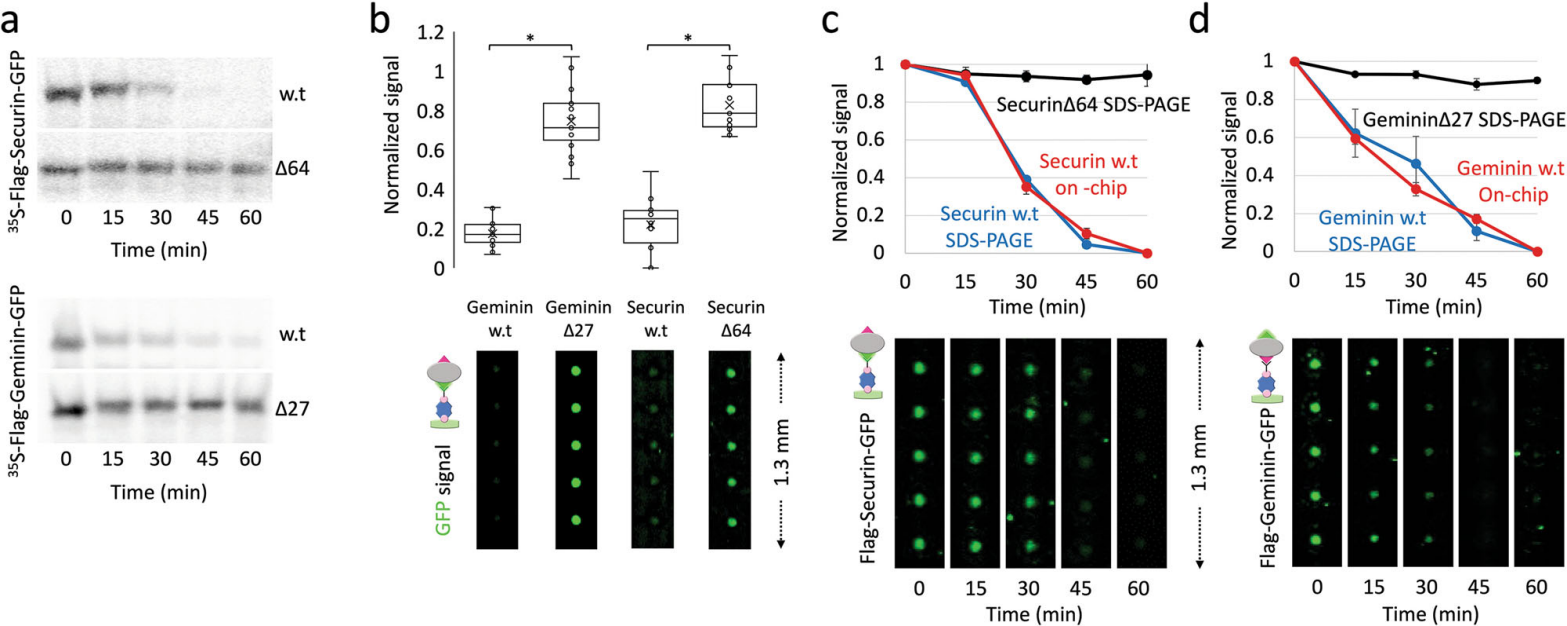
On-chip analysis of protein degradation assays. **a** Time-dependent degradation of ^35^S-labeled Flag-Securin-GFP, Flag-Geminin-GFP and their non-degradable variants (∆64 and ∆27, respectively) in NDB mitotic extracts (20 µl) supplemented with E-mix and Ubiquitin. Time-dependent proteolysis was resolved by SDS-PAGE and autoradiography. **b** Equivalent assays were performed with non-radioactive IVT products. Target proteins were incubated 1 h in reaction solution containing either extracts or PBS (control). Aliquots of each reaction mix (5 µl) were then loaded directly on a chip via separate channels, each containing dozens of cell units. Target proteins were immobilized to protein chambers via biotinylated anti-GFP antibody. The GFP tag was also used for quantification. GFP signals were calculated from 14–19 cell units per target protein per reaction condition (extracts vs. PBS). Box plots depict ratios of GFP signals (extracts/PBS) at *t*_60 min_. Mean (x) and median (-) are indicated. *p value < 0.001. Representative raw data depicting detection on chip of the four target proteins are shown. **c** The degradation of Flag-Securin-GFP variant was assayed in tube as described in B. Here, however, aliquots were snap-frozen every 15 min. Time-lapse samples were then loaded on the chip for analysis. Target proteins were immobilized in protein chambers via anti-GFP antibodies and quantified based on GFP fluorescence. Time-dependent degradations of w.t vs. mutant Securin were quantified based on ^35^S signal (standard analysis; *n* = 3] and GFP fluorescence (on-chip analysis). Plots depict mean signals and standard error bars. Mean signals were calculated from 26 cell units and normalized between max (1, *t*_0_) and min (_0_, *t*_60 min_) values, allowing proper comparison between two very different methods of detection. Error bars are shown. **d** Equivalent experiment to (c) performed with Flag-Geminin-GFP, except that immobilization was based on anti-Flag antibodies. *n* = 30–40 cell units.

Conceptually, analysis of protein degradation by pDOC is direct, simple and fast; signal detection is based on in situ quantification, thus obviating gel-electrophoresis and any other gel-related procedures, e.g: fixation, drying, autoradiography or immunoblotting, and exposure. Our first goal was to examine whether the signal sensitivity and dynamic range of pDOC enables time-based quantification of IVT products whose degradation in cell-free extracts was assayed in tube, i.e off-chip. As a proof of concept, we utilized mitotic extracts from HEK293 cells that are blocked in an anaphase-like state due to high levels of non-degradable Cyclin-B1. This mitotic cell-free system, hereafter referred to as NDB, recapitulates APC/C^Cdc20^-mediated proteolysis of the cell cycle proteins Securin and Geminin^17,35^. Conventional degradation assays of radiolabeled Flag-Securin-GFP and Flag-Geminin-GFP (IVT products) in NDB mitotic extracts are shown in Fig. 2a (see also Fig. S1). Control experiments with non-degradable mutant variants (Geminin ∆27 and Securin ∆64) demonstrate the specificity of the assay. Equivalent non-radioactive IVT products were assayed in a similar fashion. First, we performed an end-point assay (Fig. 2b). After 60 min incubation with NDB mitotic extracts, reaction samples were loaded on pDOC through separate channels and scanned for GFP fluorescence. Control reactions, in which IVT products were incubated in PBS, allowed us to normalize the level of each target protein at *t*_60 min_ (extracts/PBS ratio) and to estimate background signals. Overall, on-chip detection demonstrates a sharp reduction in the level of Geminin and Securin following incubation in NDB mitotic extracts, whereas non-degradable variants remained stable, exhibiting ~80% of the control GFP signals in PBS. At this juncture, we noted that background signals from reticulocyte lysate, cell extracts, and non-specific immobilization were minor (Fig. S2).

Next, we tested whether pDOC can be utilized to obtain reliable kinetic information on protein degradation. Flag-Securin-GFP and Flag-Geminin-GFP were incubated in NDB mitotic extracts for 60 min, and reaction samples were snap frozen in liquid nitrogen every 15 min. After quick thawing, samples representing five time-points were loaded on the chip for signal quantification. Comparable analyses were performed using SDS-PAGE and autoradiography. Non-degradable Flag-Securin∆64-GFP and Flag-Geminin∆27-GFP variants were also assayed by autoradiography for control. Considering the vast differences between the two methods of detections, GFP and autoradiography signals were normalized between 0 and 1, meaning that the signal at *t*_60 min_ was subtracted from all other time points. The resulting values were normalized to max signal at *t*_0_ and plotted. As shown in Fig. 2c, d, on-chip analysis by pDOC and off-chip analysis by SDS-PAGE-autoradiography exhibited near identical degradation patterns of Flag-Securin-GFP and Flag-Geminin-GFP. Note that reaction cocktails contained 1 μl IVT, 20 μl extracts and 2 μl ubiquitin/E. mix solution, following our standard protocol^17,27,36^. For each time point, 5 μl reaction mixes were flowed for a period of 5 min through protein chambers with open MITOMI button valves, allowing immobilization of the target protein, while the neck valves were closed. This loading protocol enabled clear visualization of the target protein without the concern of signal saturation (Fig. S3). We concluded that pDOC facilitates both end-point and time-course analyses of protein degradation in vitro. Importantly, Flag-Securin-GFP was immobilized to protein chambers via biotinylated anti-GFP antibodies rather than anti-Flag antibodies. By doing so, we effectively demonstrated that GFP can serve for both immobilization and detection, eliminating the need for two tags. Overall, we find GFP to be an optimal tag for signal detection on-chip.

**Fig. 3:**
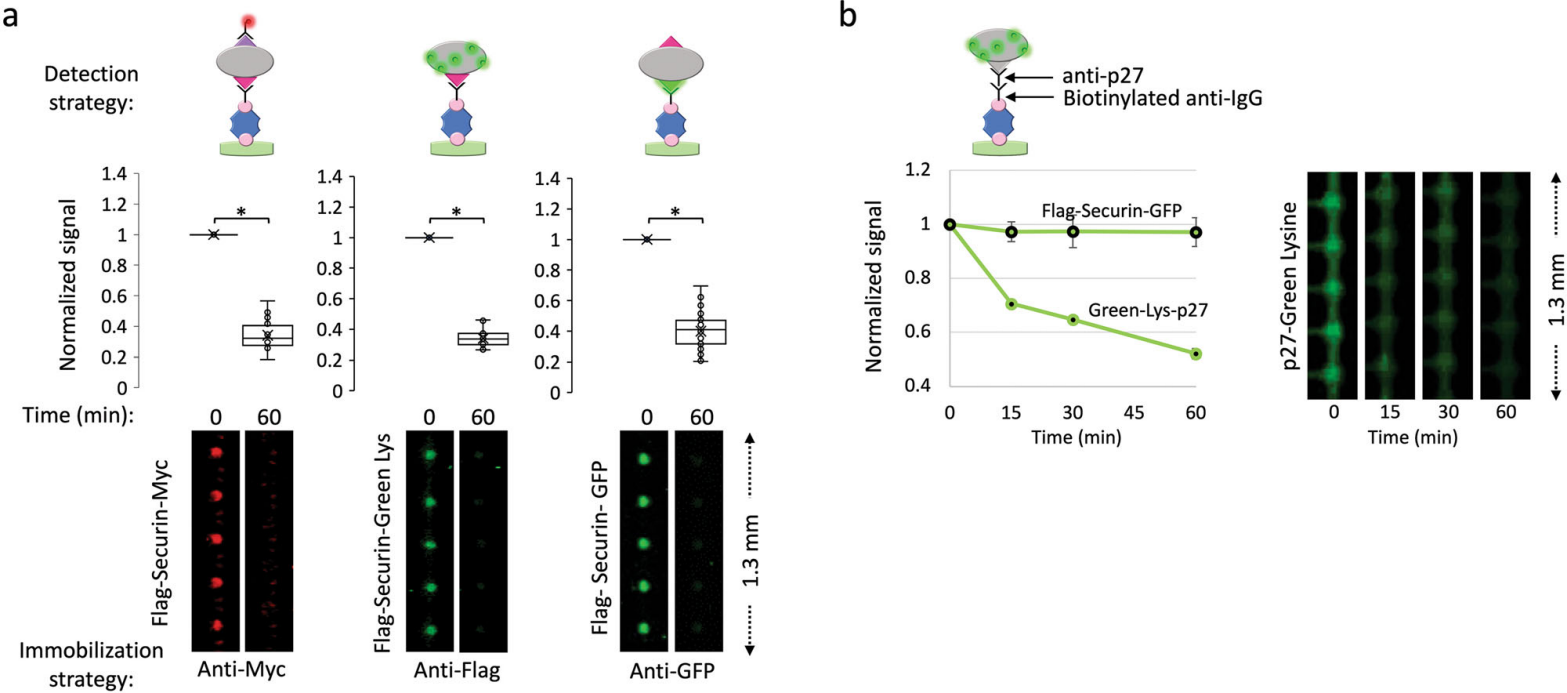
Method versatility. **a** Degradation of Flag-Securin-Myc, Flag-Securin-GFP, and Green-Lys-labeled Flag-Securin (IVT products) in NDB mitotic extracts was assayed in tube for 1 h. On-chip analysis was performed in multiple ways: 1) Flag-Securin-Myc was immobilized by anti-Myc antibodies and detected by anti-Flag Cy5-conjugated antibodies; 2) Flag-Securin-GFP was immobilized and detected via the GFP tag; and 3) Green-Lys-labeled Flag-Securin was immobilized via anti-Flag antibodies and detected by the Green-Lys signal. Anti-Myc/Flag/GFP antibodies are biotinylated. Plots and raw data depict protein levels at *t*_0_ vs. *t*_60 min_. Signals are normalized to max values at *t*_0_. Mean values and standard error bars are shown; *n* = 20–40 cell units. *p value < 0.001. **b** Degradation of Green-Lys-labeled p27 (untagged) and Flag-Securin-GFP was assayed in S-phase extracts and analyzed by pDOC. p27 was immobilized via biotinylated anti-mouse IgG and anti-p27 antibodies. The Green-Lys signal was used for detection. Flag-Securin-GFP was immobilized via biotinylated anti-Flag antibodies. Following incubation with cell extracts, protein levels were quantified by GFP or Green-Lys fluorescence in 15 min intervals. Plots depict mean and standard error values normalized to max signal at t_0_. *n* = 20 (p27) and 10 (Securin) cell units.

The fusion of a fluorescent protein to a target protein, however, may distort protein folding in ways that effectively limit proteolysis. Thus, protein quantification independent of fluorescent protein tags can, in some instances, be important. In this context, the flexibility of pDOC is particularly advantageous. Figure 3a depicts two alternative configurations by which Securin degradation could be recorded on-chip: 1) direct detection by green-Lys; and 2) indirect detection by in situ immunofluorescence. Securin level was measured at *t*_0_ and *t*_60 min_. Both approaches were informative, revealing the degradation of Securin in NDB mitotic environment, unequivocally. With respect to direct detection, it is noteworthy that IVT products tagged with GFP are, overall, brighter than equivalent proteins labeled with green-Lys. De facto, however, this experiment demonstrates that integrated green-Lys does not block proteolysis that is based of chemical modification of Lys residues^37^. Indirect detection by immunofluorescence inherently relies on two tags (single tag cannot be used for both immunolabeling and immobilization), and requires an additional incubation of 30 min. However, the small size of standard immunodetectable tags minimizes the risk of protein misfolding and the bright signal emitted from commercial fluorophores to which antibodies are coupled is advantageous.

The versatility of pDOC was further demonstrated using tag-free p27 (Fig. 3b). Degradation of p27 is mediated by SCF^Skp2^ E3 ligase, rather than APC/C, and is orchestrated with the DNA synthesis (S) phase of the cell cycle^38^. p27 degradation was assayed in extracts from S-phase synchronous HeLa S3 cells and analyzed by pDOC. The protein was immobilized via anti-p27 and biotinylated anti-IgG antibodies, and detected by green-Lys. Analysis by pDOC revealed the stereotypical instability of p27 in S-phase extracts (Fig. 3b). The degradation pattern resembled that measured by autoradiography (Fig. S4). Thus, pDOC can be utilized for degradation assays of untagged proteins. Furthermore, the method is specific and not restricted to a certain type of cell-free systems.

The advantages of autoradiography-based degradation assays include high signal-to-noise ratio, high specificity of the signal and linearity of the assay. However, this does not come without a cost. First, the ^35^S-isotope is a short-lived reagent with half-life of ~3 months. Second, on the gel the signal is spread over a well of 4–5 mm width (standard 10-well gel). Third, in a standard degradation assay, 1–2 μl IVT product is diluted in 20–30 μl cell extracts whose protein concentration is about 20–25 mg/ml. Thus, the amount of IVT loaded per lane is limited by the maximum separation capacity of the gel. Typically, we load 4–5 μl of reaction mix into a well of a standard 10-well mini-gel of 1 mm thick. Fourth, in vitro translation is challenging for large proteins because of ribosome processivity. Thus, although large proteins incorporate more radiolabeled Methionine/Cysteine relative to small proteins, the overall signal of the full-length protein can be impractical for reliable quantification. Fifth, the exposure time needed for a high-quality signal is usually within a range of 12–24 h. Note that abovementioned points 2–4 are equally relevant when protein degradation is assayed by SDS-PAGE and immunoblotting. As for point 5, immunoblotting does not require long exposures. Yet, the overall incubation time with antibodies is long. Furthermore, IVT products in immunoblot-based assays must be tagged in order to be distinguished from the endogenous proteins in the extracts. At this juncture, it is important to note that whether degradation is assayed by autoradiography or immunoblotting, informative expression of IVT products must be validated beforehand, in itself, a day long procedure. Equivalent validation by pDOC is instantaneous.

**Fig. 4:**
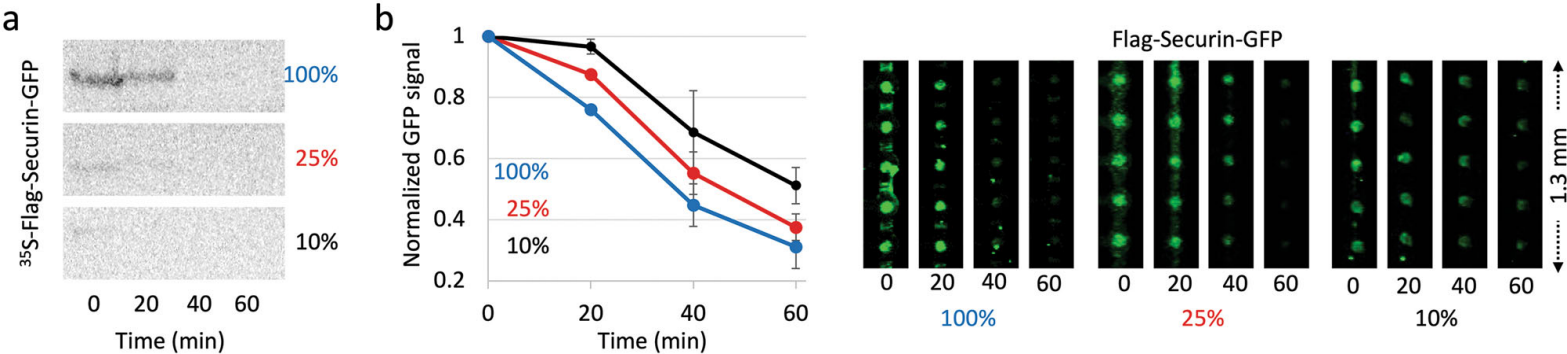
Method sensitivity. **a** Time-dependent degradation assay of ^35^S-labeled Flag-Securin-GFP in NDB mitotic extracts. Assays were performed with undiluted IVT product (100%) or following 4x/10x dilution in reticulocyte lysate (25 and 10%, respectively). In all assays, 1 µl substrate was incubated in 20 µl cell extracts. Samples were snap-frozen in 20 min intervals and assayed by SDS-PAGE and autoradiography. **b** Equivalent degradation assays performed with non-radioactive Flag-Securin-GFP. Time-point samples were loaded on the chip for immobilization (via anti-GFP-biotinylated antibody) and detection (GFP fluorescence). The plots summarize data from three experiments, 15–20 cell units per experiment. Normalized mean and standard error values are shown (left). Representative raw data are shown on the right.

On-chip, the fluorescence signals of Flag-Securin-GFP at *t*_60 min_ were above background level and more noticeable compared to autoradiography (Figs. 2a and 3a). Yet, when the signal at *t*_60 min_ was subtracted from all other time-points, the overall degradation patterns of Flag-Securin-GFP, as revealed by pDOC and autoradiography, was similar (Fig. 2c, d). This observation suggests that pDOC detects protein residue still lingering in the reaction mix after 60 min incubation, and which are barely detected by autoradiography, if at all. Thus, it can be argued that the sensitivity of pDOC surpasses that of the conventional autoradiography, and if so, pDOC not only facilitates in vitro degradation assays, but also significantly reduces reagent consumption and cost per assay. To test that, we diluted Flag-Securin-GFP in reticulocyte lysate 4- and 10-fold and incubated the substrate in NDB mitotic extracts for 1 h, while maintaining the original reticulocyte lysate /extract volume ratio of 1/20 (µl). Time point samples were analyzed by pDOC (based on GFP fluorescence). Equivalent experiments performed in parallel with ^35^S-labeled Flag-Securin-GFP, following the conventional assay (Figs. 4 and S5). To clarify, radiolabeled and non-radiolabeled substrates were expressed simultaneously from the same TNT®/DNA solution mix. Furthermore, degradation assays performed a day after delivery of the ^35^S-Met/^35^S-Cys solution to our lab (~1175 Ci/mmol), and the gels were exposed to phosphor screen overnight. Yet, whenever the IVT substrate was diluted, the signal obtained by autoradiography was below any acceptable standard, even at *t*_0,_ and decreased to barely or undetectable levels after 15 min incubation (Figs. 4a and S5). Conversely, analyses by pDOC were informative in all three conditions (Fig. 4b). We could detect bona fide signals of 4- and 10-fold diluted Flag-Securin-GFP at *t*_0_ as well as at *t*_60 min_, recording the full dynamics of the protein in NDB mitotic extracts. On a more practical note, we effectively demonstrated that protein degradation can be analyzed with 0.1 µl IVT product and 2 µl cell extracts, thereby saving 90% of the reagents. This feature is particularly valuable in assays which rely on limited biological material, e.g., extracts from primary cells, and normal/pathological tissue samples.

pDOC unveils remanent amount of Flag-Securin-GFP that could not be visualized by conventional methods. Interestingly, while the overall degradation pattern of Flag-Securin-GFP in all three conditions was similar, we noticed a systematic delay in Flag-Securin-GFP degradation as the substrate concentration was reduced. This observation has not been identified previously in our lab, and could be attributed to rate-limiting steps along the process of ubiquitination/degradation of Securin, and perhaps APC/C substrates overall^39,40^. Note that the estimated concentration of Flag-Securin-GFP in the reaction mix was 17 nM (before further dilution in reticulocyte lysate; Fig. S6).

### On-chip assay for protein degradation

**Fig. 5:**
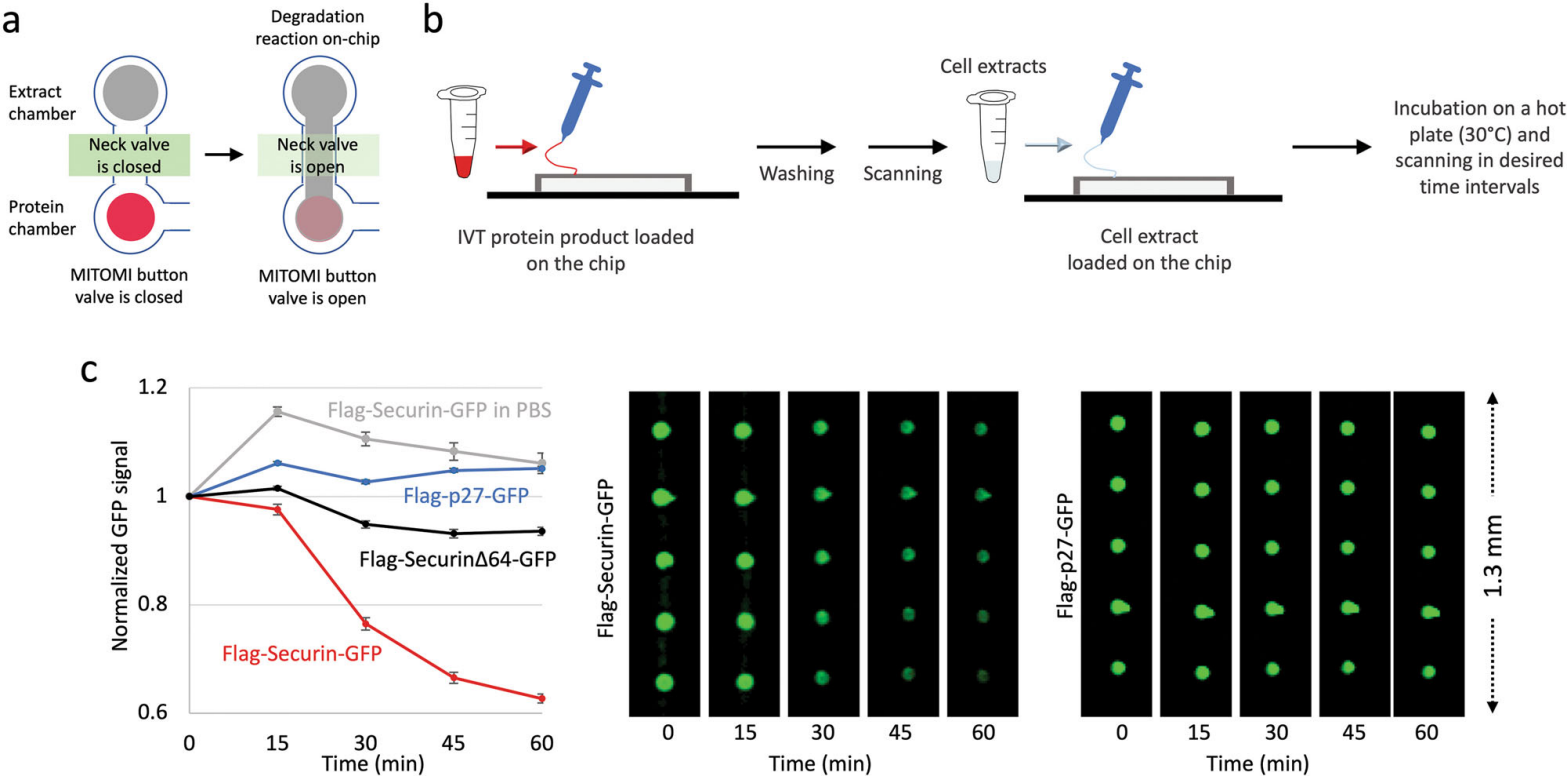
Protein degradation on chip. **a**, **b** Schematic illustration of complete on-chip degradation assay by pDOC. IVT protein products are immobilized to the surface of the ‘protein chamber’ via anti GFP biotinylated antibody (see more information in Fig. 1). The closing of the MITOMI button valve traps the protein. All remains are washed away with PBS. Proper expression and immobilization of the target proteins are validated by scanning. Next, cell-free extracts are loaded into the extract chamber and trapped by closing the neck valve. The reaction begins with the opening of neck and MITOMI button valves, which allows diffusion of cell extracts into the ‘protein chamber’ and mixing with the target protein. The chip is placed on a 30 °C hot plate and scanned at 15 min intervals to provide kinetic information in real time. **c** GFP-tagged Securin and p27 IVT products were immobilized via anti-GFP-biotinylated antibody. Extract chambers were then filled with NDB mitotic extracts that support ubiquitination of Securin, but not of its non-degradable variant (Δ64) and p27 (negative control validating assay specificity). Protein degradation was assayed for 1 h during which the chip was scanned five times. The plot depicts mean GFP signals normalized to *t*_0_ and standard error bars; *n* = 14–30 cell units. Representative raw data for p27 and Securin are shown on the right.

Until now, we have demonstrated the capacity of pDOC to facilitate analysis of protein degradation reactions, which were performed in tube, i.e. off-chip (Figs. 2–4). Technically, however, neck and MITOMI button valves allow time-regulated mixing between the target protein and cell-free extracts within each cell unit. More specifically, loading and immobilization of target proteins are performed with closed neck valves. Once target proteins are trapped under closed MITOMI buttons, cell extracts can be flown into the chip. This step is performed with open neck valves, allowing cell extracts to reach chamber I. Cell extracts can be then trapped in chamber I, noted here as ‘extract chamber’, by reclosing the neck valve, and all remaining materials are then washed away. Degradation reaction commences (*t*_0_) with the opening of both neck and MITOMI button valves. Cell extracts diffuse into the protein chamber and reach the exposed target protein (illustrated in Fig. 5a). Potentially, this pipeline enables a complete on-chip assay for protein degradation. The motivation for an on-chip assay is threefold: 1) reagent-saving and cost per assay. In fact, 5 μl cell extracts are sufficient to fill a thousand cell units; 2) analysis of protein degradation in real time. 3) higher throughput, especially if the target proteins are expressed on-chip, as was previously demonstrated^25–27^. The challenge, however, is the limited degradation capacity of <1 nl extracts per cell unit, which never been tested on any platform. We decided to test the feasibility of protein degradation on chip. To this end, wt and non-degradable variants of Flag-Securin-GFP as well as Flag-p27-GFP (IVT products) were loaded on the chip (through separate channels) and captured in protein chambers underneath the MITOMI button valve via biotinylated anti-GFP antibody. After washing, NDB mitotic extracts were loaded into the extract chamber and trapped by closing the neck valve. Untrapped materials were washed away. The device was heated to 30 °C and scanned to obtain signals of *t*_0_. The opening of neck and the MITOMI button valve initiated degradation reactions in all cell units simultaneously (Fig. 5a, b), and the chip was scanned in time intervals of 15 min. Whereas the fluorescent signal of non-degradable Securin, p27, as well as GFP itself (Fig. S7), remained stable in NDB mitotic extracts throughout the experiment, the signal of Flag-Securin-GFP diminished with time (Fig. 5c), revealing the regulated proteolysis of this protein in mitotic environment.

**Fig. 6:**
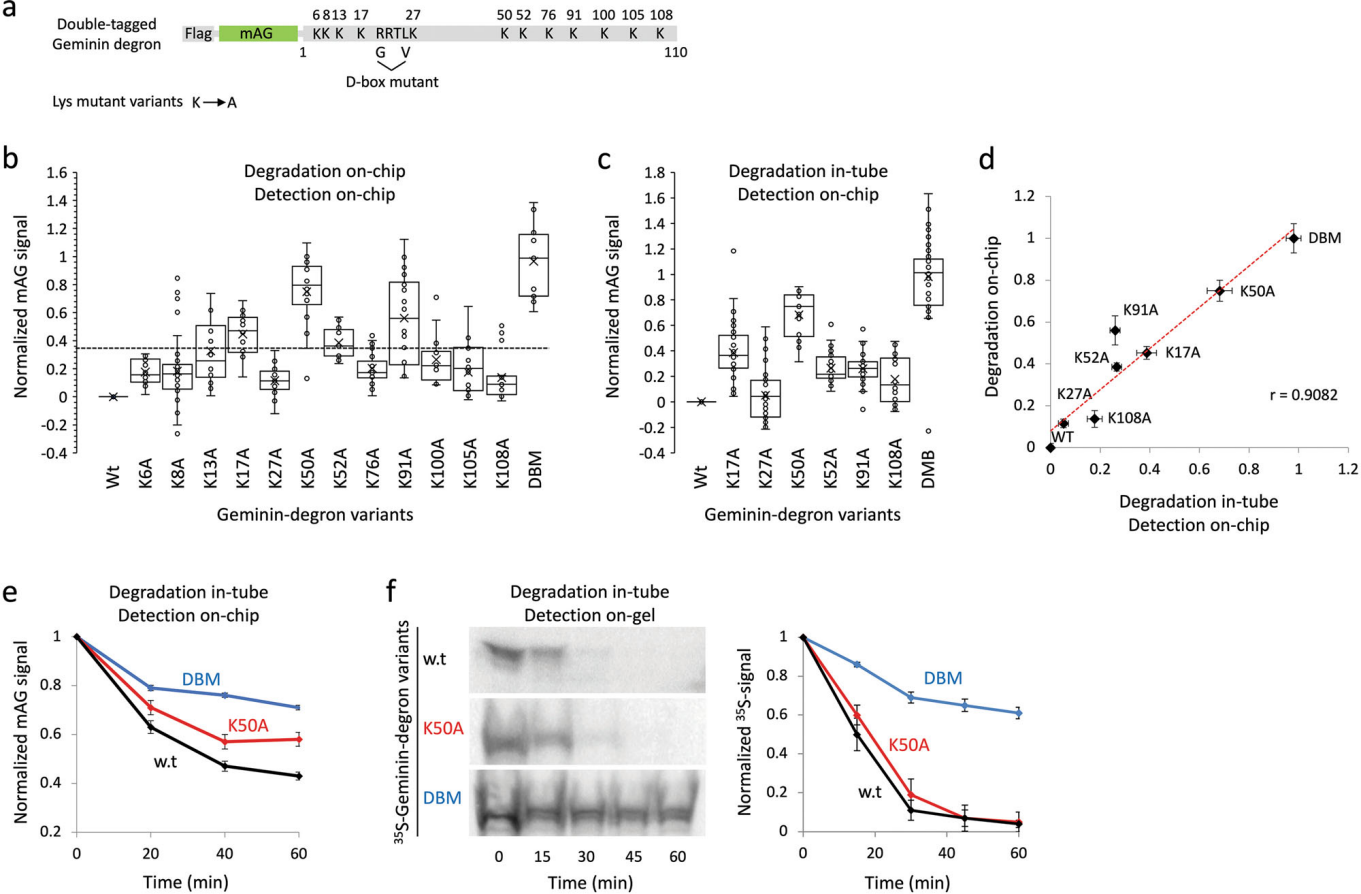
Analysis by pDOC uncovers limited degradation of K50A Geminin mutant in mitotic environment. **a** An illustration of Geminin-degron tagged with Flag and mAG. Lysins and the core element (RxxL) of the destruction box (D-box) are indicated by single-letter amino acid code. Mutant variants were generated by substituting single lysine residues with alanine. D-box mutant (DBM) Geminin carries glycine and valine at positions 23 and 26, respectively. **b** End-point degradation analysis of Geminin-degron variants on chip. Dashed line represents a cut-off value (i.e., four standard deviations of w.t Geminin-degron at *t*_0_) above which variants were flagged for further analyses. **c** End-point degradations analysis of selected Geminin-degron variants. Degradation reactions were performed in tube and protein levels were quantified on chip (see Fig. 2 for technical details). **b**, **c** Normalized levels of Geminin-degron variants at *t*_60 min_ are plotted. *n* = 12–38 (b) and 21–57 (c) cell units. **d** A linear correlation between the data depicted in B and C. Mean and standard error values are shown. **e**, **f** Time-dependent degradations of Geminin-degron variants (in-tube) were analyzed on chip (e; *n* = 16–25 cell units) by SDS-PAGE and autoradiography (f; Representative raw data are shown on the left; *n* = 3). All degradation reactions are in NDB mitotic extracts.

Lysine is the primary site for ubiquitination and dozens of other PTMs that may regulate protein degradation in less straightforward manners. We took advantage of the multiplexing capability of pDOC and tested the contribution of lysine residues to the overall degradation of Geminin in mitotic environment. The cis-elements regulating the degradation of Geminin are located at the protein’s N-terminus. In fact, monomeric Azami-Green (mAG) fluorescent protein attached to Geminin’s first 110 amino acids (mAG-Geminin) is a well-known marker for APC/C activity in cells^31,41^. We constructed a Flag-tagged mAG-Geminin, hereafter will be referred to as ‘Geminin-degron’, and generated a panel of mutant variants in which single lysine (K) residues were substituted with alanine (A). A non-degradable variant, in which the destruction box sequence ^23^RxxL^26^,^42^, was substituted to GxxV (single amino acid code), was also constructed (Fig. 6a). The degradation of all variants in NDB mitotic extracts was first assayed on-chip (see Fig. 5 for more technical details). IVT products were immobilized to the chip via Biotinylated anti-Flag antibodies and quantified by mAG fluorescence at *t*_0_ and *t*_60 min_. The signal ratio between these time points was normalized between 0 and 1, i.e., the ratio calculated for wt (0) and non-degradable (1) Geminin-degrons (Fig. 6b). Next, a subset of mutant variants were subjected to off-chip degradation reactions followed by an on-chip analysis (Fig. 6c; see Fig. 2 for more technical details). A similar degradation trend was observed in both end-point assays (Fig. 6d). Note, the same strategy used for signal quantification and normalization. Importantly, the two assays exhibited a limited degradation capacity for the K50A variant in NDB mitotic extracts. The partial stability of the K50A variant was further validated by time-dependent degradation analysis (Fig. 6e). To this end, in-tube degradation reactions were sampled every 20 min and analyzed on-chip. Interestingly, the limited degradation or K50A variant could not be detected by SDS-PAGE and autoradiography (Figs. 6f and S8). This discrepancy can be explained by differential sensitivity of the two methods and the capacity of pDOC to detect lesser amounts of substrate (Fig. 4). Taken together, our findings suggest that lysine 50 contributes to the degradation of Geminin. At this present, it is unclear whether lysine 50 is polyubiquitinated. But if we were to follow this model, ubiquitination of Geminin is clearly not restricted to lysine 50 or else K50A variant would have remained stable in NDB mitotic extracts. More generally, the data presented in Fig. 6 demonstrate systematic analysis of protein degradation by pDOC.

## Discussion

**Fig. 7:**
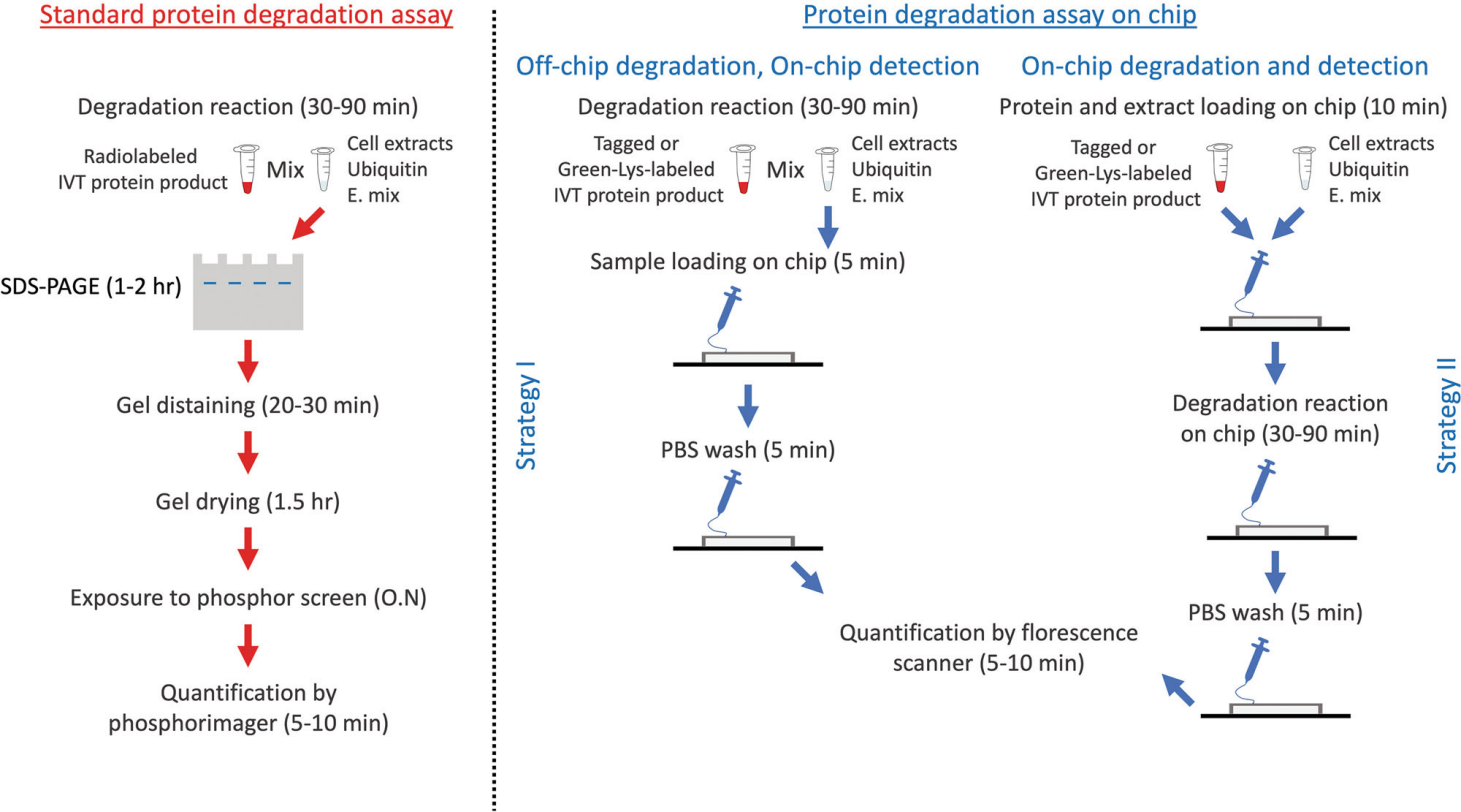
Schematic illustration of standard vs. two on-chip assays for protein degradation. The purpose of all methods is to assay protein degradation of IVT protein products in reaction mix comprising cell-free extracts, recombinant ubiquitin and energy-regeneration mixture (E. mix). The standard assay (red pipeline) is typically radioactive. An ^35^S-labeled IVT product (1–2 µl) is mixed with 20–50 µl reaction mixture, and incubated for 30–90 min. Each time point an aliquot (~5 µl) of the reaction mix is harvested. Samples are denatured and resolved by SDS-PAGE. Following drying, the gel is exposed to a phosphor screen. Usually, a satisfactory signal is obtained overnight (O.N). Longer exposures (24–72 h) are often needed when the radioactive signal is low due to a variety of reasons. pDOC provides two alternative assays (blue pipelines), both with benefits. Strategy I: Degradation reactions are performed off-chip. The target protein is either fused to a standard tag (e.g., GFP, Flag, Myc, etc.) or translated in the presence of Green-Lys. Time-point samples are either loaded on the chip sequentially or boiled/snap-frozen first and then loaded simultaneously. The target protein is immobilized by tag/protein-specific surface antibodies, all other materials are washed away, and quantification is carried out by one of the strategies detailed in Fig. 1c almost instantly. Strategy II: Here, target proteins and cell extracts are loaded on the chip successively and occupy separate chambers within each cell unit. The mixing of the two martials initiates the reaction. Time-lapse scanning provides kinetic information. While Strategy I is simpler, Strategy II is more optimal for high-throughput experiments. Both on-chip strategies are reagent-saving and considerably shorter than the standard assay.

pDOC is a lab-on-chip platform devised to facilitate and simplify discovery and analysis of protein degradation in physiologically relevant contexts. The chip accommodates hundreds of microchambers in which protein degradation can be assayed promptly and simultaneously. It is important to emphasize, however, that the motivation and need for pDOC far transcends its high-throughput potential: 1) analyses can be performed with considerably lower quantities of reagents compared to conventional assays. The latter feature is especially crucial for cell-free extracts whose production can be a bottleneck in terms of time, activity, and amount, e.g., cell extracts whose origin is precious normal/malignant tissue samples for the purpose of global/personal biomedical research/diagnostics; 2) Signal detection is based on in situ quantification of fluorescent signal. The method is independent of contaminating radioactive materials and yet, with sensitivity that surpasses traditional assays. A comparison between pDOC and the conventional degradation assay is illustrated in Fig. 7) the flexibility of pDOC is substantial; the platform facilitates almost all possible experimental designs. First, both tagged and untagged proteins can be assayed, with detection based on the incorporation of Green-Lys, fluorescent proteins, or immunodetectable tags. Second, pDOC can be used as an integrated microfluidics column for instant analyses of low-volume off-chip degradation reactions (Figs. 2–3). The integration of two loading modules allows simultaneous loading of dozens of reaction solutions on the chip. Target proteins are purified and concentrated on the protein chamber of the cell units, allowing on-chip detection of off-chip degradation within minutes to 1 h (depending on the detection protocol). Alternatively, protein degradation can be assayed entirely on-chip and in real time. This feature of pDOC is important not only because of the ability to test protein degradation in sub-nanoliter volume but because of the inherent compatibility of MITOMI-based devices with on-chip in vitro translation^24,25,27^. By expressing array of proteins on-chip, one can multiplex target proteins from tens to hundreds, increasing throughput dramatically. The downside of on-chip expression is that the assembly of the device with the DNA microarray is not trivial and currently has to be performed by experts in specialized laboratories. Overall, the multiplex nature of pDOC is bilateral: one reaction solution can be applied to dozens or hundreds of different proteins, or one or many proteins can be tested for degradation in multiple physiochemical conditions, simultaneously. This quality is relevant for the analysis of both on-chip and off-chip degradation reactions.

Ubiquitin-mediated proteolysis is routinely assayed in hundreds of laboratories worldwide. Furthermore, it is a major signaling route for therapy (e.g Velcade®) and drug design (e.g., Nurix Therapeutics). We devised pDOC to facilitate and simplify in vitro analyses of protein degradation in quasicellular environments. The method is fast, sensitive, reagent-saving, and amenable for both low- and high-throughput studies. It is also noteworthy that full automation of the platform is foreseeable. We therefore believe that pDOC is a valuable tool for basic and translational research of targeted protein degradation.

### Reporting summary

Further information on research design is available in the Nature Research Reporting Summary linked to this article.

## Supplementary information

Supplementary Information

Description of Additional Supplementary Files

Reporting Summary

## Data Availability

Data generated or analyzed during this study are included in this published article (and its supplementary information files). Source data for Figures and Supplementary figures are provided in two.xlsx files: Supplementary data 1 (Figures) and Supplementary data 2 (Supplementary Figures).
