# Supplementary material for: Affinity microfluidics enables high-throughput protein degradation analysis in cell-free extracts
Source: Commun Biol. 2022 Oct 28;5:1147. doi: 10.1038/s42003-022-04103-3 (PMC9616816; doi:10.1038/s42003-022-04103-3)
Supplement: Supplementary file 1 — Supplementary Information [file 42003_2022_4103_MOESM1_ESM.pdf]

Supplementary information

for

Affinity microfluidics enables high-throughput protein degradation analysis in cell-free extracts

Lev Brio[1,^], Danit Wasserman[1,^], Efrat Michaely-Barbiro[1], Gal Barazany-Gal[1], Doron Gerber[1,§,*], and Amit Tzur[1,§,*]

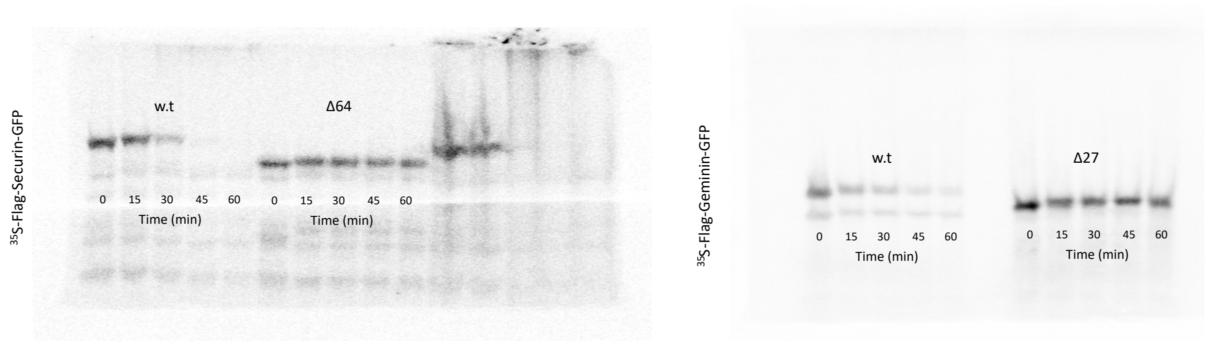

Figure S1. Unedited gel image for Figure 2a.

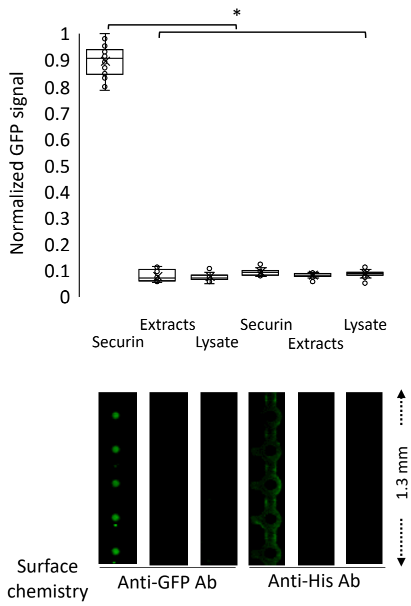

Figure S2. Estimating background and noise signals on chip. An IVT product of Securin-GFP was loaded on a chip, immobilized to protein chambers via biotinylated anti-GFP antibodies, and mixed (on chip) with NDB mitotic extracts (see more information in figures 1 and 6). The GFP signal of the protein was immediately measured. Control experiments performed with 1) NDB mitotic extracts without Securin-GFP (Extracts) to measure the 488 nm-excited autofluorescence of the extracts; and 2) NDB mitotic extracts with reticulocyte lysate to measure the contribution of the lysate to the overall signal of the target protein. A similar set of experiments performed following surface chemistry with biotinylated anti-His antibodies to measure the contribution of non-specific protein interactions to the overall signal coming from the protein chamber. Box plot depicts mean (x) and median (-) signals normalized to maximum level. *n*=20-40; **p* value <0.01. Representative raw data are shown.

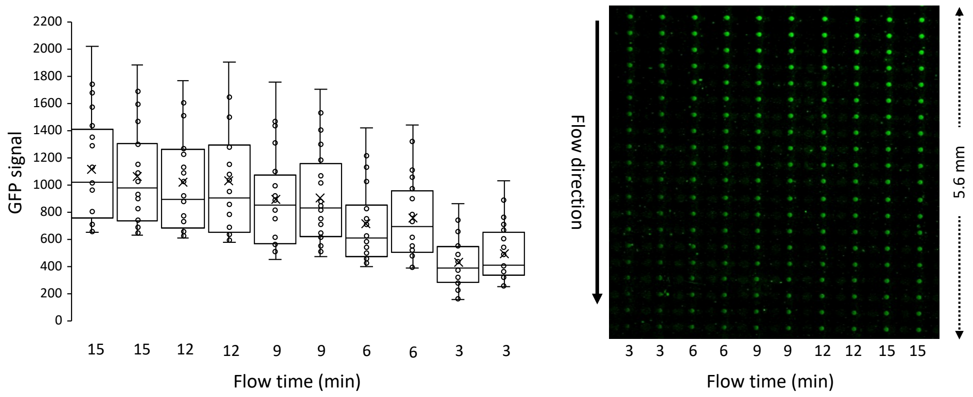

Figure S3. Validating unsaturated signal detection by pDOC. 1 µl IVT product of non-degradable (Δ64) Flag-Securin-GFP was mixed with 20 µl NDB mitotic extracts, following the volume ratio of a standard degradation reaction. Samples were immediately flowed on the pDOC device via separate channels (two chanels per sample) in increasing time duration of 3 to 15 minutes. Flag-Securin-GFP was immobilized to protein chambers via biotinylated anti-GFP antibodies and detected by GFP fluorescence. The plot depicts mean values (x) calculated from 20 cell units. Signal units are arbitrary.

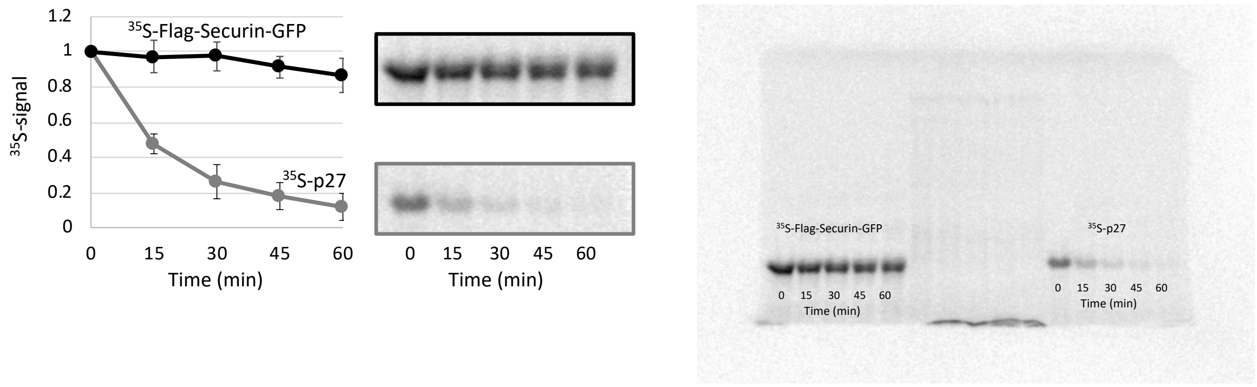

Figure S4. Conventional degradation assay of p27. During DNA synthesis (S) phase, p27 is ubiquitinated by the SCF^{Skp2} E3 complex and degraded whereas Securin remain stable. ^{35}S-labeled IVT products of both proteins were incubated in human cell-free system recapitulating S-phase. Protein degradation was assayed by SDS-PAGE and autoradiography (standard protocol). p27, but not Securin, was degraded in this condition. Quantifications and representative raw data are shown. Unedited gel image is shown on the right. The plot depicts mean and standard error values; *n*=3.

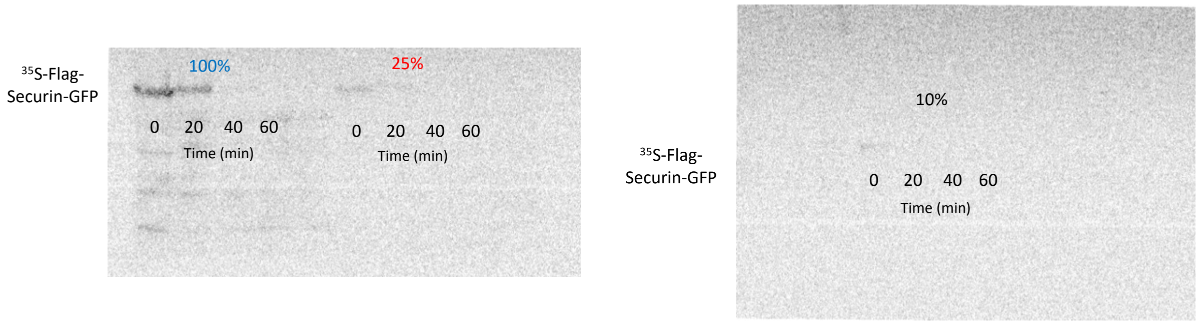

Figure S5. Unedited gel image for Figure 4a.

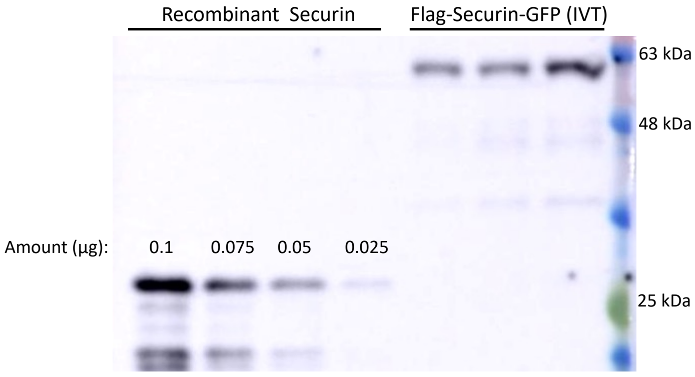

Figure S6. An estimation of Securin concentration in reticulocyte lysate following translation. Various amounts of reombinant His-Securin [36] and 1 µl of three Flag-Securin-GFP IVT products were resolved on SDS-PAGE for Western blot analysis with anti-Securin antibody. The estimated concentration of Flag-Securin-GFP IVT product is 400 nM.

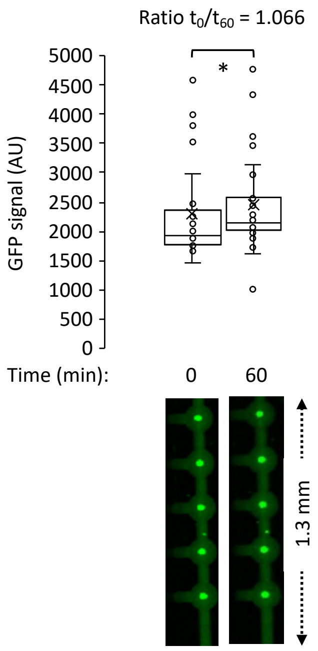

Figure S7. On-chip degradtion of GFP. An IVT product of GFP was incubated with NDB mitotic extracts on chip. Biotynylated anti-GFP antibodies were used for immobilization. GFP signal was recorded at time 0 and 60 min, demostarteing the stability of GFP in NDB mitotic extracts. Box plot depicts mean (x) and median (-) signals. n=26/31; *p value =0.33 Representative raw data are shown. See Figure 5 for more technical details on on-chip degradation.

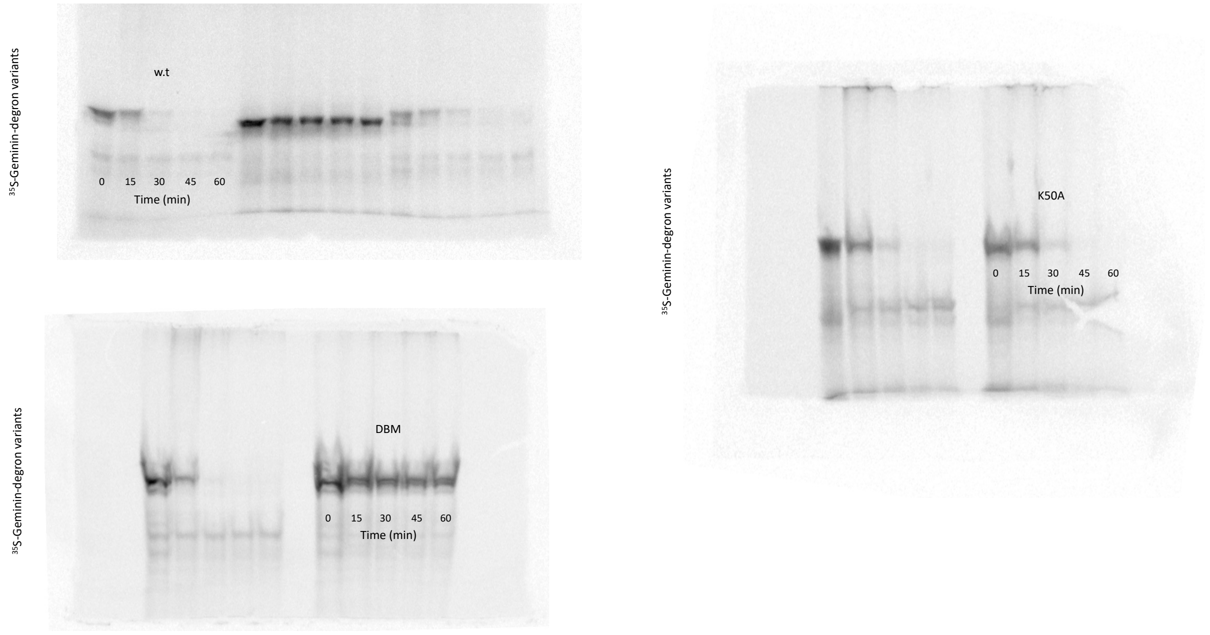

Figure S8. Unedited gel image for Figure 6f.

Supplementary Table 1. A list of primers used for deletion and site-directed mutagenesis of Geminin variants

Mutant	Primer Fw	Primer Rev
Δ27	GGCGGCCGCTCGAGATGATTCAGCCTTC	GAAGGCTGAATCATCTCGAGCGGCCGCC
K6A	GCTCGAGATGAATCCCAGTATGGCGCA GAAACAAGAAGAAATCAAA	TTTGATTTCTTCTTGTTTCTGCGCCATACTGG GATTCATCTCGAGC
K8A	CGAGATGAATCCCAGTATGAAGCAGGC ACAAGAAGAAATCAAAGAGAATATA	TATATTCTCTTTGATTTCTTCTTGTGCCTGCTT CATACTGGGATTCATCTCG
K13A	GTATGAAGCAGAAACAAGAAGAAATCG CAGAGAATATAAAGAATAGTTCTGTCC	GGACAGAACTATTCTTTATATTCTCTGCGATT TCTTCTTGTTTCTGCTTCATAC
K17A	AAACAAGAAGAAATCAAAGAGAATATA GCGAATAGTTCTGTCCCAAGAAGAACTC	GAGTTCTTCTTGGGACAGAACTATTCGCTATA TTCTCTTTGATTTCTTCTTGTTT
K27A	TGTCCCAAGAAGAACTCTGGCGATGATT CAGCCTTCTGCA	TGCAGAAGGCTGAATCATCGCCAGAGTTCTT CTTGGGACA
K50A	CTGTCCGCAGGCTTGTCCGCAAGGAAAC ATCGGAATGA	TCATTCCGATGTTTCCTTGCGGACAAGCCTGC GGACAG
K52A	CGCAGGCTTGTCCAAAAGGGCACATCG GAATGACCACTTA	TAAGTGGTCATTCCGATGTGCCCTTTTGGACA AGCCTGCG
K76A	GTCCCAGAATCTAGTGAAAATGCAAATC TTGGAGGAGTCACCC	GGTGACTCCTCCAAGATTTGCATTTTCACTAG ATTCTGGGAC
K91A	CACCCAGGAGTCATTTGATCTTATGATT GCAGAAAATCCATCCTCTCA	TGAGAGGATGGATTTTCTGCAATCATAAGAT CAAATGACTCCTGGGTG
K100A	TCCATCCTCTCAGTATTGGGCGGAAGTG GCAGAAAAACGG	CCGTTTTTCTGCCACTTCCGCCCAATACTGAG AGGATGGA
K105A	ATTGGAAGGAAGTGGCAGAAGCACGGA GAAAGGCGCTG	CAGCGCCTTTCTCCGTGCTTCTGCCACTTCCT TCCAAT
K108A	AGGAAGTGGCAGAAAAACGGAGAGCGG CGCTGTAAGG	CCTTACAGCGCCGCTCTCCGTTTTTCTGCCAC TTCCT
D-box mutant R23G and L26V	TATAAAGAATAGTTCTGTCCCAGGAAGA ACTGTGAAGATGATTCAGCCTTC	GAAGGCTGAATCATCTTCACAGTTCTTCCTGG GACAGAACTATTCTTTATA
