# Supplementary material for: Affinity microfluidics enables high-throughput protein degradation analysis in cell-free extracts
Source: Commun Biol. 2022 Oct 28;5:1147. doi: 10.1038/s42003-022-04103-3 (PMC9616816; doi:10.1038/s42003-022-04103-3)
Supplement: Supplementary file 2 — Description of Additional Supplementary Files [file 42003_2022_4103_MOESM2_ESM.pdf]

Description of Additional Supplementary Files

File name: Supplementary Data 1
Description: Source data for figures 2-6

File name: Supplementary Data 2
Description: Source data for supplementary figures s1-s5
